# Activation of RNase L in Egyptian Rousette Bat-Derived RoNi/7 Cells Is Dependent Primarily on OAS3 and Independent of MAVS Signaling

**DOI:** 10.1128/mBio.02414-19

**Published:** 2019-11-12

**Authors:** Yize Li, Beihua Dong, Zuzhang Wei, Robert H. Silverman, Susan R. Weiss

**Affiliations:** a Department of Microbiology, Perelman School of Medicine, University of Pennsylvania, Philadelphia, Pennsylvania, USA; b Department of Cancer Biology, Cleveland Clinic, Cleveland, Ohio, USA; Indiana University Bloomington

**Keywords:** OAS-RNase L, bat innate immunity, *Rousettus aegyptiacus*, Egyptian Rousette, dsRNA-induced pathways, OAS-RNase

## Abstract

Bats are reservoirs for many RNA viruses that are highly pathogenic in humans yet relatively apathogenic in the natural host. It has been suggested that differences in innate immunity are responsible. The antiviral OAS-RNase L pathway is well characterized in humans, but there is little known about its activation and antiviral activity in bats. During infection, OASs, upon sensing double-stranded RNA (dsRNA), produce 2′-5′ oligoadenylates (2-5A), leading to activation of RNase L which degrades viral and host RNA, limiting viral replication. Humans encode three active OASs (OAS1 to -3). Analysis of the Egyptian Rousette bat genome combined with mRNA sequencing from bat RoNi/7 cells revealed three homologous OAS proteins. Interferon alpha treatment or viral infection induced all three OAS mRNAs, but RNase L mRNA is constitutively expressed. Sindbis virus (SINV) or vaccinia virus (VACVΔE3L) infection of wild-type (WT) or *OAS1-*KO (knockout), *OAS2*-KO, or *MAVS-*KO RoNi/7 cells, but not *RNase L*-KO or *OAS3*-KO cells, induces robust RNase L activation. SINV replication is 100- to 200-fold higher in the absence of RNase L or OAS3 than in WT cells. However, *MAVS*-KO had no detectable effect on RNA degradation or replication. Thus, in RoNi/7 bat cells, as in human cells, activation of RNase L during infection and its antiviral activity are dependent primarily on OAS3 while MAVS signaling is not required for the activation of RNase L and restriction of infection. Our findings indicate that OAS proteins serve as pattern recognition receptors (PRRs) to recognize viral dsRNA and that this pathway is a primary response to virus rather than a secondary effect of interferon signaling.

## Abstract (precis)

Many RNA viruses that are highly pathogenic in humans are relatively apathogenic in their bat reservoirs, making it important to compare innate immune responses in bats to those well characterized in humans. One such antiviral response is the OAS-RNase L pathway. OASs, upon sensing dsRNA, produce 2-5A, leading to activation of RNase L which degrades viral and host RNA, limiting viral replication. Analysis of Egyptian Rousette bat sequences revealed three OAS genes expressing OAS1, OAS2, and OAS3 proteins. Interferon treatment or viral infection induces all three bat OAS mRNAs. In these bat cells as in human cells, RNase L activation and its antiviral activity are dependent primarily on OAS3 while MAVS signaling is not required. Importantly, our findings indicate the OAS-RNase L system is a primary response to virus rather than a secondary effect of interferon signaling and therefore can be activated early in infection or while interferon signaling is antagonized.

## INTRODUCTION

Bats are reservoirs for many RNA viruses that are highly pathogenic in humans yet attenuated in their natural host. These include filoviruses (Ebola virus and Marburg virus) (1–4), coronaviruses (severe acute respiratory syndrome coronavirus [SARS-CoV] and Middle East respiratory syndrome coronavirus [MERS-CoV]) (5, 6), alphaviruses (Venezuelan equine encephalitis virus [VEEV]) (7), and Nipah/Hendra viruses (8). Several studies have suggested that bats can tolerate infection due to an enhanced innate immune response that enables early control of infection and prevents systemic dissemination (9–14). For example, three alpha interferon (IFN-α) genes are constitutively expressed in black flying fox, Pteropus alecto (12). Other studies report that bats have a dampened host response, speculated to promote virus-host coexistence (15, 16). For example, the cGAS-STING pathway is dampened in some bats species due to a mutation in STING (15) and the inflammasome DNA sensor AIM2 is missing from almost all the known bat species (17). Thus, there is a need for further investigation into the innate immune response in bats and how it impacts viral infection.

Double-stranded RNA (dsRNA)-induced innate immune responses play a critical role in limiting viral infection (18). One dsRNA-induced and potent antiviral pathway is the OAS-RNase L system, which has been well characterized in human cells and murine cells (19). The human OAS family contains four members, three enzymatic active proteins (OAS1, OAS2, and OAS3) and one OAS-like (OASL) protein, lacking enzymatic activity (20). All three enzymatically active OASs contain a core unit with dsRNA binding and catalytic functions (21, 22). OAS2 and OAS3 duplicate one or two nonenzymatic units which are believed to enhance the binding affinity to dsRNA. Mice express homologous OAS proteins that produce 2′-5′ oligoadenylates (2-5A), including OAS1a/g, OAS2, and OAS3, as well as OASL2 and several catalytically inactive OAS isoforms, OASL1, and additional OAS1 proteins (23, 24).

After sensing dsRNA, the catalytic domain of OASs undergoes a conformational rearrangement to form the catalytic cavity and, from ATP, synthesizes 2-5A. 2-5A binds to monomeric RNase L, leading to dimerization and activation to cleave viral and cellular single-stranded RNAs, thereby blocking viral replication as well as protein synthesis (25). While all three OASs (OAS1 to -3) produce 2-5A upon binding dsRNA *in vitro* or when overexpressed (21), we showed previously, using a series of cells with OAS gene knockouts (KOs), that only OAS3 was required for detectable activation of RNase L during infection of three human cell lines with diverse viruses (26).

There is scant information in the scientific literature about activation or inhibition of the OAS-RNase L pathway in bats and its contributions to bat innate immunity, although it was shown that OAS-RNase L can be activated by poly(rI)·poly(rC) (pIC) in *P. alecto* bat cells (27). We screened a group of bat cells (discussed further below) for activation of RNase L during Sindbis virus (SINV) infection and chose to carry out further studies in Egyptian Rousette (ER) bat (Egyptian fruit bat)-derived RoNi/7 cells. Through bioinformatic analysis of the annotated genomic sequence of Egyptian Rousette bats from GenBank (28), we identified three OAS genes (*bOAS1*, *bOAS2*, and *bOAS3*) and two OASL genes (*bOASL1* and *bOASL2*). (We designate bat genes or proteins as bOAS to differentiate them from human [h] or mouse [m] genes or proteins.) We utilized CRISPR-Cas9 gene-editing technology to generate bOAS-KO Egyptian Rousette-derived RoNi/7 cells (29). We found that, as in human cells, the activation of RNase L is dependent on bOAS3 expression during infection with SINV. In addition, KO of *bOAS3* or *RNASEL* leads to greater than 100-fold more SINV replication, while KO of either *bOAS1* or *bOAS2* promotes more modest increases in replication. As in human cells, activation of RNase L by SINV is independent of MAVS expression in bat RoNi/7 cells, suggesting that RNase L may be activated either before or without virus-induced IFN. Similar results were obtained with vaccinia virus, VACVΔE3L, in bat RoNi/7 cells. Finally, our data indicate that the OAS-RNase L pathway has a greater antiviral effect than MAVS-dependent IFN signaling in RoNi cells as well as human cells.

## RESULTS

### The bat genome contains three Oas genes with interferon-stimulated response elements (ISREs) in the promoter region, two Oasl genes, and one RNase L gene.

**FIG 1:**
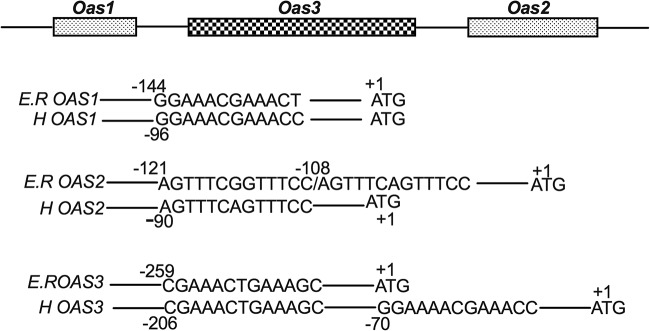
Interferon-stimulated response elements (ISREs) in the Egyptian Rousette (ER) *Oas1*, *Oas2*, and *Oas3* genes. Bat OAS ISRE sequences were compared to those in human (H) OAS genes. The numbers indicate the positions of the ISREs relative to each ATG initiation codon (+1). The gene sequences were derived from GenBank: *bOas1* (ID 107513273), *bOas3-*like (ID 107513228) for both *Oas2* and *Oas3* ISREs, *hOAS1* (ID 4938), *hOAS2* (ID 4939), and *hOAS3* (ID 4940).

Using the genomic sequences of Egyptian Rousette bat from GenBank, we analyzed the genes and protein sequences of bat OASs, OASLs, and RNase L (28). The genes were annotated as *OAS1* (GenBank ID 107513273), *OAS3-*like (GenBank ID 107513228), *OASL* (GenBank ID 107513228), and *OASL2* (GenBank ID 107501264). No *OAS2* was noted. The Egyptian Rousette (ER) *bOAS1* gene encodes a protein that shares 67% and 59% amino acid sequence similarity to human *OAS1* and mouse *Oas1*, respectively (Table 1). The annotated ER *OAS3-*like gene encodes a protein of 1,903 amino acids. Amino acids 1 to 1082 share 80% similarity with human OAS3 (1,083 amino acids), and amino acids 1197 to 1903 share 71% similarity with human OAS2 (709 amino acids). These results suggested that the annotated ER *OAS3*-like gene encoded an OAS3-OAS2 fusion protein. Since in most known species, *OAS2* and *OAS3* are two separate genes, we could not exclude the possibility that ER *OAS3* and *OAS2* genes were falsely annotated as one gene. To clarify this question, we attempted to clone the mRNA encoded by the *bOAS1* and *bOAS3*-like genes. We cloned the *bOAS1* open reading frame (ORF) successfully, but we could not clone the mRNA predicted to encode a bOAS3-bOAS2 fusion protein. Instead, we cloned two cDNAs which encode bOAS2 and bOAS3 open reading frames separately. These results suggested that ER bats and humans share similar OAS gene structures (Fig. 1).

**TABLE 1:** Amino acid sequence analysis of bat human and mouse OAS and OAS-like proteins

Protein		% similarity^*b*^				
Name	Catalytic activity^*a*^	hOAS1/mOAS1a	hOAS2/mOAS2	hOAS3/mOAS3	hOASL/mOASL1	mOASL2
bOAS1	Yes	67/59	ND^*c*^	ND	ND	ND
bOAS2	Yes	ND	71/62	ND	ND	ND
bOAS3	Yes	ND	ND	80/71	ND	ND
bOASL1	No	ND	ND	ND	79/77	59
bOASL2	Yes	37/33	ND	ND	56/54	69

a Protein catalytic activity was predicted by the conservation in the active site of the P-loop and aspartic acid triad.

b Amino acid consensus similarity. GenBank protein accession numbers are shown in parentheses as follows: bat proteins, bOAS1 (XP_016005252), bOAS2 (QCT83250.1), bOAS3 (QCT83251.1), bOASL1 (XP_015982708), and bOASL2 (XP_015982728); human proteins, hOAS1 (NP_058132.2), hOAS2 (NP_058197.2), hOAS3 (NP_006178.2), and hOASL1 (Q15646); mouse proteins, mOAS1a (P11928), mOAS2 (E9Q9A9), mOAS3 (Q8VI93), mOASL1 (Q8VI94), and mOASL2 (Q9Z2F2). Vector NTI 10 was used to do the sequence alignment.

c ND, not determined.

Human and mouse OAS genes are induced by interferon (IFN) (i.e., they are interferon-stimulated genes [ISGs]) and thus contain interferon-stimulated response elements (ISREs) in the promoter regions, required for interferon-inducible transcription. To determine whether ER Oas genes are ISGs, we analyzed the genomic sequences for ISREs (A/GNGAAANNGAAACT or AGTTTCNNTTTCNC/T) (30). We found that the ER *Oas1* gene promoter region contains one ISRE with high sequence identity with that of human *OAS1* (Fig. 1). Upstream of the bOAS2 coding region, we found two adjacent ISREs in the opposite polarity, the first with homology to the one ISRE found in the human *OAS2* gene. The ISRE of ER *bOAS3* is identical to the first one of two found in the human *OAS3* gene. We did not find an ISRE in the promoter region of the ER RNase L gene.

### bOAS1, bOAS2, and bOAS3 mRNAs are induced by IFN-α or infection with SINV or SeV in RoNi/7 cells.

**FIG 2:**
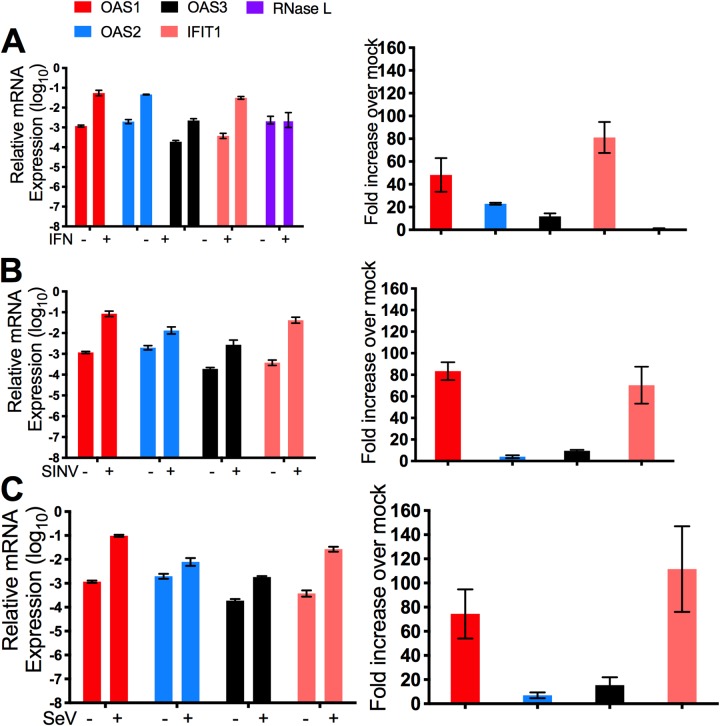
Induction of OAS1, OAS2, OAS3, and IFIT1 mRNA expression in RoNi/7 cells, following treatment with IFN-α or viral infection. RoNi/7 cells were treated with 1,000 U of human universal IFN-α (A) or infected with SINV (B) or SeV (C) (MOI = 10), cells were lysed at 24 h posttreatment or 12 (SINV) or 24 (SeV) h postinfection, and RNA was isolated. The mRNA levels were determined by qRT-PCR, calculated relative to β-actin mRNA, and expressed as fold over levels of mock treatment using the formula 2^−Δ(Δ^*^CT^*^)^ (Δ*C_T_* = *C_T_*_gene of interest_ − *C_T_*_actin_). The data are pooled from three independent experiments.

RoNi/7 cells were treated with human universal IFN-α, the relative mRNA levels were determined by quantitative reverse transcriptase PCR (qRT-PCR), and the fold increase in expression over mock-treated cells was calculated. Induction of bat *IFIT1* (ISG56) mRNA was used as a positive control (14) and was increased by 81-fold upon IFN-α treatment (Fig. 2A). All three bOAS mRNAs were induced by IFN-α; the bOAS1 mRNA level increased by 48-fold, while the mRNA level of bOAS2 and bOAS3 increased by 22- and 11-fold, respectively (Fig. 2A), consistent with the ISREs in their promoters. The mRNA level of RNase L did not change upon IFN treatment in RoNi/7 cells, indicating that, similarly to the human *RNASEL* gene, bat *RNASEL* is not an ISG (Fig. 2A), consistent with the lack of an ISRE in its promoter region (data not shown). To investigate induction of bOAS genes during viral infection, RoNi/7 cells were infected with SINV or Sendai virus (SeV). While the relative mRNA level of bOAS1 increased by 70- to 80-fold during SINV or SeV infection, bOAS2 mRNA was only weakly (4- to 6-fold) induced and bOAS3 mRNA was induced somewhat more (15- or 9-fold) during SINV or SeV infection, respectively (Fig. 2B and C), similar to induction by IFN-α (Fig. 2A to C).

### Amino acid sequence alignment indicates that the Egyptian Rousette bOAS1, bOAS2, bOAS3, and bOASL2 proteins are oligoadenylate synthetases with potential catalytic activity.

The human and mouse OAS genes and proteins have been extensively studied (21, 24). To investigate the bat OAS gene family, we aligned the predicted amino acid sequences of bat, human, and mouse OAS and OASL proteins. While bOAS protein sequences are highly homologous to human and mouse proteins, in each case bOAS1, bOAS2, and bOAS3 share more homology with human than with mouse proteins (Table 1).

**FIG 3:**
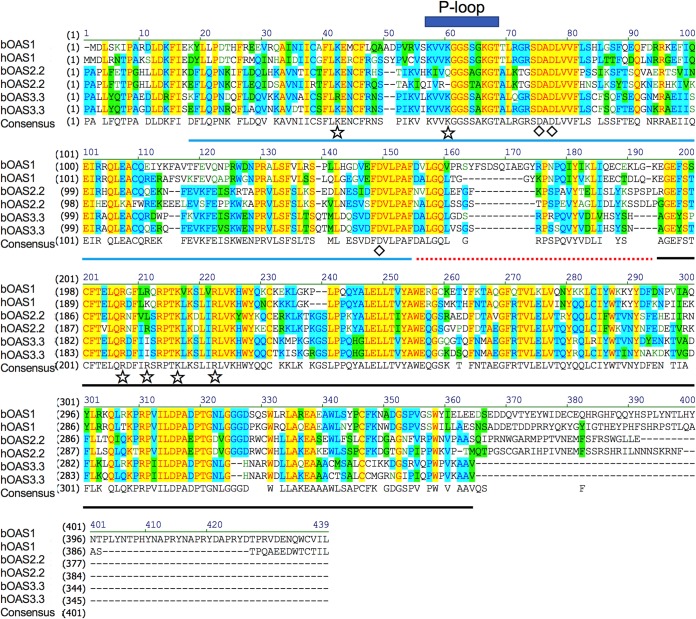
Sequence alignment of Egyptian Rousette (ER) OAS1, OAS2, and OAS3 catalytic domains with human OAS sequences. ER full-length OAS1 (XP_016005252) and the C-terminal catalytic domain of OAS2 and OAS3 (translated from mRNA sequencing of cDNA clones) were aligned with the corresponding portions of human OAS1 p46 (NP_058132.2), OAS2 p69 (NP_058197.2), and OAS3 p100 (NP_006178.2). Blue line, N lobe of the catalytic domain; red dotted line, the linker sequences of N and C lobes; black line, C lobe of the catalytic domain; diamonds, catalytic aspartic acids; stars, residues binding to dsRNA; blue rectangle, P-loop. Background color: yellow, identical; blue, conserved; green, similar; white, nonsimilar.

We aligned the catalytic domain of each bOAS protein (full-length bOAS1, domain II of bOAS2 [bOAS2.2], and domain III of bOAS3 [bOAS3.3]) with the corresponding protein or domains of human OAS1, OAS2, and OAS3 (Fig. 3). Based on sequence homology and previous studies of the structure of human (31) and porcine (32) OAS1, bat OAS catalytic domains are composed of three parts, the N-terminal lobe (blue line in Fig. 3), the linker (red dotted line), and the C-terminal lobe (black line). Three aspartic acid residues (Asp75, Asp77, and Asp148 of hOAS1) indicated with diamonds are essential for enzymatic function of OASs and conserved among all the bat and human OASs (33). The P-loop which contributes to the formation of the catalytic cavity is also conserved. The Arg or Lys residues (indicated by stars) participate in dsRNA binding and are conserved among all bat or human OASs. These results predict that bOAS1, bOAS2.2, and bOAS3.3 have the potential to recognize dsRNA and synthesize 2-5A.

Besides three Oas genes, the Egyptian Rousette genome has two OAS-like genes (*OASL1* and *OASL2*). The bOASL1 protein shares high sequence similarity with hOASL (79%) and mOASL1 (77%), suggesting that, like these human and mouse proteins, it does not have oligoadenylate synthetase activity (Table 1; see also Fig. S1 in the supplemental material). However, bOASL2 is similar to mOASL2 (69%) (Table 1) and when aligned with human OAS1 has a conserved catalytic domain, suggesting that it may have oligoadenylate synthetase activity (Fig. S2).

10.1128/mBio.02414-19.1 FIG S1 Amino acid sequence alignment of bat and mouse OASL1 with human OASL. ER OASL1 (XP_015982708) is aligned with mouse OASL1 (Q8VI94) and human OASL (Q15646). Download FIG S1, TIF file, 1.2 MB. Copyright © 2019 Li et al. 2019 Li et al. This content is distributed under the terms of the Creative Commons Attribution 4.0 International license.

10.1128/mBio.02414-19.2 FIG S2 Amino acid sequence alignment of bat and mouse OASL2 with human OAS1. ER OASL2 (XP_015982728) is aligned with mouse OASL2 (Q9Z2F2) and human OAS1 (NP_058132.2). Diamonds, catalytic aspartic acids; blue rectangle, P-loop. Download FIG S2, TIF file, 0.8 MB. Copyright © 2019 Li et al. 2019 Li et al. This content is distributed under the terms of the Creative Commons Attribution 4.0 International license.

### Egyptian Rousette (ER) RNase L shares high amino acid similarity to human RNase L with conserved 2-5A binding sites and endoribonuclease catalytic sites.

**FIG 4:**
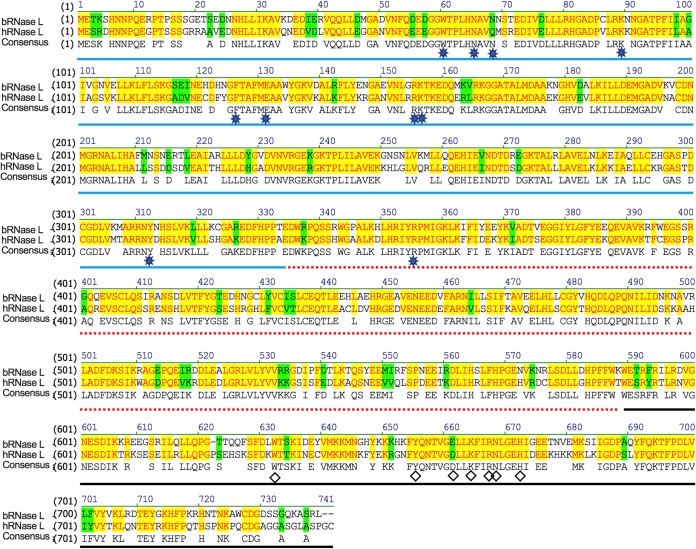
Amino acid sequence alignment of Egyptian Rousette (ER) with human RNase L indicates high conservation of the protein. ER RNase L (XP_016001031.1) was aligned with human RNase L (Q05823.2). Blue line, ankyrin-repeat domain; red dotted line, pseudokinase (PK) domain; black line, RNase domain; diamonds, conserved residues in the catalytic domain; stars, residues binding to 2-5A. Background color: yellow, identical; green, similar; white, nonsimilar.

By sequence alignment, we found that bRNase L shares 77% amino acid similarity with the human protein (Fig. 4). Similar to human or porcine RNase L, bRNase L contains three predicted domains, an N-terminal ankyrin-repeat domain (blue line in Fig. 4), a pseudo-protein kinase domain (red dotted line), and an RNase domain (black line) (34). The amino acids which are responsible for 2-5A binding (stars) and catalysis residues (diamonds) are conserved among bat and human RNase L sequences.

### Protein expression levels of OAS2 and OAS3 in bat cells are upregulated by IFN treatment.

**FIG 5:**
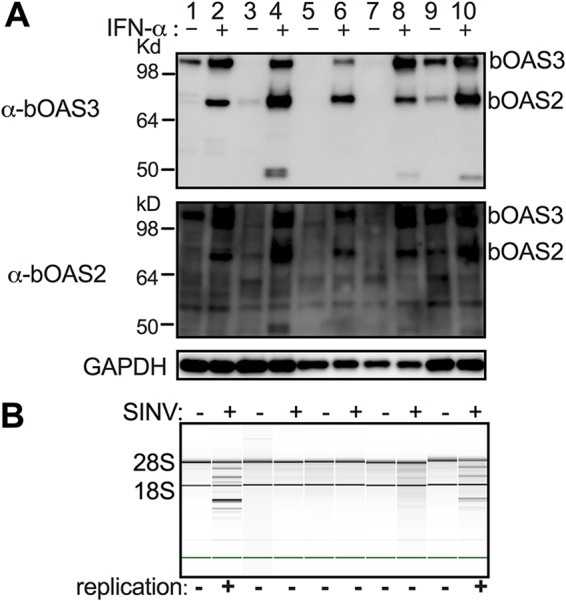
Expression of bOAS2 and bOAS3 proteins is induced by IFN-α treatment in cells derived from multiple bat species, and RNase L is activated during infection of RoNi/7 cells and Eptesicus fuscus skin fibroblasts. Bat cells (lanes 1 and 2, RoNi/7 cells; lanes 3 and 4, Myotis lucifugus skin fibroblasts; lanes 5 and 6, Myotis lucifugus embryonic fibroblasts; lanes 7 and 8, *Myotis velifer* embryonic fibroblasts; lanes 9 and 10, Eptesicus fuscus skin fibroblasts) were treated with 1,000 U universal human IFN-α for 24 h and lysed, and proteins were analyzed by polyacrylamide gel electrophoresis and Western immunoblotting with rabbit polyclonal antibodies to bOAS2 or bOAS3 (A), or cells were infected with SINV (MOI = 10 PFU/cell) and, at 12 h postinfection, lysed, and rRNA integrity was assessed by Bioanalyzer, with the positions of 18S and 28S rRNA indicated (B). Virus replication as assessed by visualization of SINV-mCherry expression in infected cells is also indicated at the bottom of the panel. The data in each panel are from one representative of two independent experiments.

We generated rabbit polyclonal antibodies against domain II of bOAS2 (anti-bOAS2) or domains II and III of bOAS3 (anti-bOAS3). (Attempts to raise antisera against bOAS1 were unsuccessful.) We used these antisera to determine both basal and induced OAS protein expression levels in bat cells derived from several species of bats. Interestingly, antisera against either bOAS2 or bOAS3 detected both OAS proteins. Basal expression of OAS3 was detected in RoNi/7 cells and Eptesicus fuscus skin fibroblasts, and the expression was upregulated upon IFN-α treatment in both cell types (Fig. 5A). While no to low basal OAS3 expression was detected in Myotis lucifugus skin fibroblasts, Myotis lucifugus embryonic fibroblasts, and Myotis velifer embryonic fibroblasts, the OAS3 protein expression level became detectable upon IFN treatment (Fig. 5A). While basal OAS2 protein expression was observed in Eptesicus fuscus skin fibroblasts and weakly in Myotis lucifugus skin fibroblasts, expression of bOAS2 was detected in all of these bat cells following IFN treatment (Fig. 5A). No OAS3-OAS2 fusion protein was detected in these cells, consistent with our cDNA cloning data. To assess activation of RNase L in these cells, we infected these bat cell lines with SINV; RNase L was activated in RoNi/7 cells and Eptesicus fuscus skin fibroblasts but not in other bat cells (Fig. 5B), likely because SINV failed to replicate.

### Activation of RNase L during SINV infection is dependent on OAS3 expression in RoNi/7 cells.

**FIG 6:**
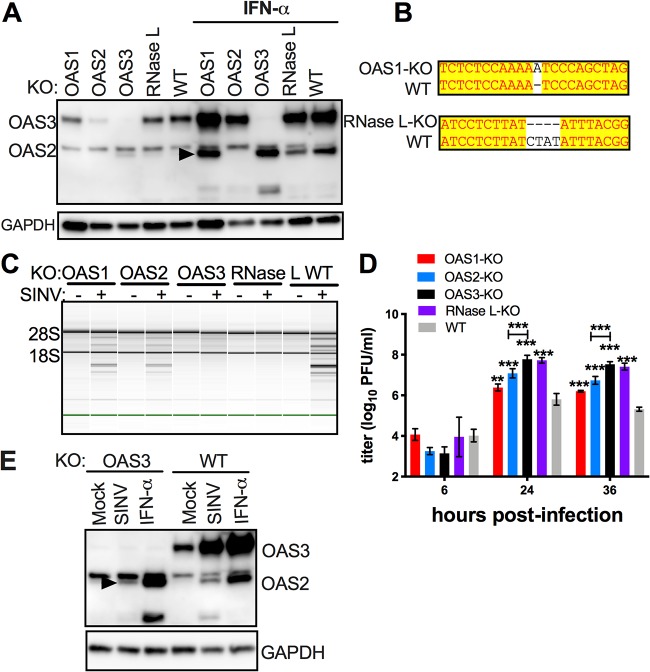
Activation of RNase L during SINV infection of RoNi/7 cells requires OAS3 expression. (A) *bOas1*, *bOas2*, *bOas3*, and *bRNase L*-KO RoNi/7 cells were mock treated or treated with 1,000 U of IFN-α overnight. Cells were lysed, and proteins were analyzed by polyacrylamide gel electrophoresis followed by Western immunoblotting with rabbit polyclonal antibodies against bOAS3. The arrowhead indicates bOAS2. The data are from one representative of two independent experiments. (B) DNA sequences encompassing the mutations from the *Oas1* gene of *bOAS1-*KO cells and the *RNase L* gene of *bRNase L-*KO cells were amplified, sequenced, and compared with the reference sequences of the genes. (C) WT and KO RoNi/7 cells were infected with SINV (MOI = 10 PFU/cell); at 12 h postinfection; cells were lysed; and RNA integrity was assessed on a Bioanalyzer. The positions of 18S and 28S rRNA are indicated. The data are from one representative of two independent experiments. (D) Cells were infected with SINV (MOI = 1 PFU/cell); at the indicated time points, the supernatants were harvested; and infectious viruses were titrated by plaque assay on Vero cells. The viral titer data are pooled from two independent experiments with three biological replicates in each experiment and expressed as means ± SDs (***, *P < *0.001). (E) Cells were infected with SINV (MOI = 1 PFU/cell) or treated with 1,000 U IFN-α; at 16 h after infection or treatment, cells were lysed; and proteins were analyzed by Western immunoblotting with rabbit polyclonal antibodies against bOAS3. The data are from one representative of two independent experiments.

We next sought to determine whether the activation of RNase L in bat RoNi/7 cells is dependent on expression of one or more OAS genes. Thus, we deployed the clustered regularly interspaced short palindromic repeat (CRISPR)-Cas9 gene-editing technology to KO expression of *bOAS1*, *bOAS2*, *bOAS3*, and *bRNase L*, each separately, from RoNi/7 cells. The KOs of *bOAS2* and *bOAS3* were confirmed by Western blotting, while the disruptions of the *OAS1* and *RNase L* genes for which we lacked antibodies were confirmed by sequencing of the CRISPR-induced insertion (*bOAS1*) or deletion (*RNase L*) (Fig. 6A and B). Upon infection with SINV, degradation of rRNA, as assessed by Bioanalyzer, was detected in wild-type (WT) and *bOAS1-*KO and *bOAS2-*KO cells but not in *bOAS3-*KO and *bRNase L-*KO cells (Fig. 6C), indicating that the activation of RNase L during SINV infection in RoNi/7 cells is dependent on bOAS3 expression, similar to our previous findings in human cells. We assessed viral replication in this set of KO cells at several time points postinfection. At 24 and 36 h postinfection (hpi), both *bRNase L*-KO and *bOAS3*-KO cells showed approximately 100- to 200-fold higher titers than the parental WT cells (Fig. 6D). Viral titers from *bOAS1*-KO and *bOAS2-*KO cells were more modestly elevated than in WT cells at 24 and 36 h postinfection, 3- to 8-fold for *bOAS1-*KO and 12- to 15-fold for *bOAS2-*KO (Fig. 6D). When the second *bOAS2-*KO and *bOAS3-*KO clones were infected with SINV, similar data were obtained for activation of RNase L by Bioanalyzer assay (Fig. S3A) and for virus replication (Fig. S3B and C). The second *bOAS2-*KO clone examined displayed lower levels of virus replication than the clone originally used, closer to levels observed in WT cells (Fig. 6), but neither clone showed increased RNase L activation as assessed by Bioanalyzer, compared to WT cells (Fig. S3). It is important to note that while bOAS1 and bOAS2 mRNA and protein expression levels are upregulated during SINV infection (Fig. 2A and Fig. 6E), this is not sufficient to activate RNase L in the absence of bOAS3 expression (Fig. 6C).

10.1128/mBio.02414-19.3 FIG S3 Comparison of RNase L activation levels of two OAS2- and OAS3-KO clones of RoNi/7 cells. WT and two *bOAS2*-KO or *bOAS3*-KO RoNi/7 clones were infected with SINV (MOI = 10 PFU/cell); at 12 hours postinfection, cells were lysed; and RNA integrity was assessed on a Bioanalyzer. The positions of 18S and 28S rRNA are indicated. The data are from one representative of two independent experiments. (B and C) WT and KO RoNi/7 clones were infected with SINV (MOI = 1 PFU/cell); at the indicated time points, the supernatants were harvested; and infectious viruses were titrated by plaque assay on Vero cells. The data are pooled from two independent experiments with three biological replicates in each experiment and expressed as means ± SDs (**, *P < *0.01; ***, *P < *0.001). (The clones labeled “#1” are the ones used to generate the data in Fig. 6.) Download FIG S3, TIF file, 0.3 MB. Copyright © 2019 Li et al. 2019 Li et al. This content is distributed under the terms of the Creative Commons Attribution 4.0 International license.

**FIG 7:**
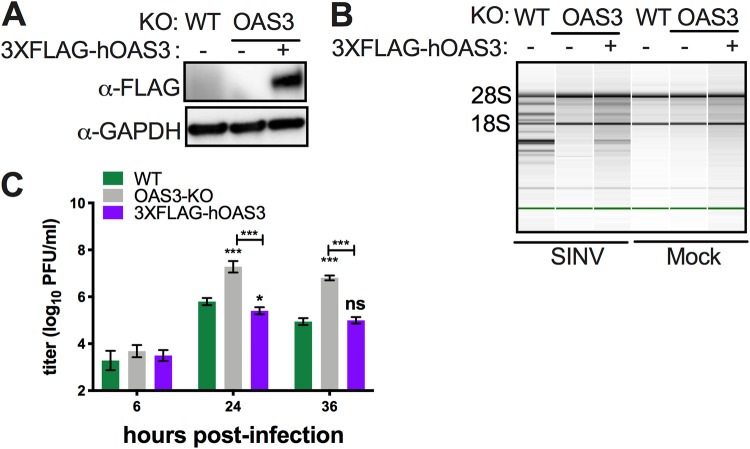
Expression of hOAS3 in *bOAS3-*KO RoNi cells restores RNase L activation. (A) *OAS3*-KO RoNi/7 cells, electroporated with pCMV10-3×FLAG-hOAS3, selected, and cloned as described in Materials and Methods, were lysed, and proteins were analyzed by Western immunoblotting with anti-FLAG monoclonal antibody. (B) WT and *OAS3*-KO cells and *OAS3*-KO cells expressing 3×FLAG-hOAS3 were infected with SINV (MOI = 10 PFU/cell); at 12 h postinfection, cells were lysed; and RNA integrity was assessed on a Bioanalyzer. The positions of 18S and 28S rRNA are indicated. The data are from one representative of two independent experiments. (C) WT and *OAS3*-KO cells and *OAS3*-KO cells expressing 3×FLAG-hOAS3 were infected with SINV (MOI = 1 PFU/cell); at the indicated time points, the supernatants were harvested; and infectious viruses were titrated by plaque assay on Vero cells. The viral titer data are pooled from two independent experiments with three biological replicates in each experiment and expressed as means ± SDs (***, *P < *0.001; ns, not significant).

To verify that OAS3-KO cells were competent to activate RNase L, we introduced FLAG-tagged human OAS3 into *bOAS3-*KO RoNi/7 cells. (We used human OAS3 rather than bat OAS because they are about 80% homologous and we did not have a full-length bOAS clone assembled.) Expression of 3×FLAG-hOAS3 in *bOAS3-*KO RoNi/7 cells was verified by Western blotting (Fig. 7A). WT, *bOAS3-*KO, and 3×FLAG-hOAS3-expressing cells were infected with SINV. Degradation of rRNA, as assessed by Bioanalyzer, was detected in WT and 3×FLAG-hOAS3 cells but, as expected, not in *bOAS3-*KO cells (Fig. 7B), confirming that these cells are competent to activate RNase L when they express OAS3. We assessed viral replication in this set of cells at several time points postinfection. At 6, 24, and 36 h postinfection, 3×FLAG-hOAS3-KO cells produced the same titers as parental WT cells, approximately 100- to 200-fold lower than *bOAS3*-KO cells (Fig. 7C), confirming as expected that hOAS3 expressed in bOAS3-KO cells restores the antiviral effects of RNase L activation and thus restricts virus replication to the same extent as in WT cells.

### MAVS expression is not required for RNase L activation and limitation of SINV infection in bat RoNi/7 and human A549 cells.

**FIG 8:**
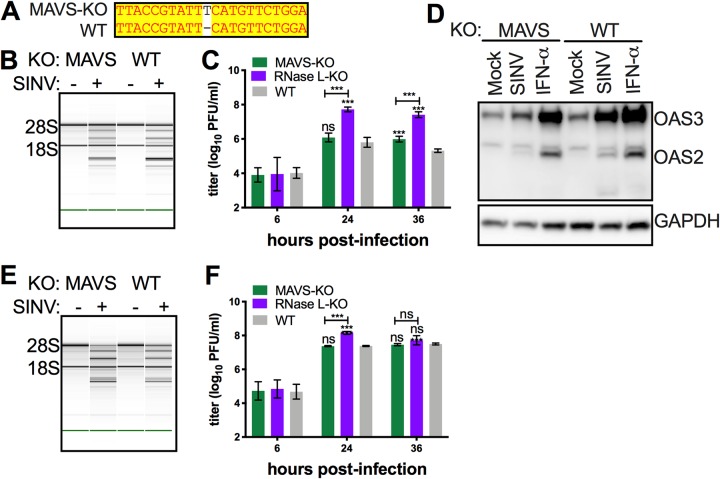
MAVS-independent activation of OAS-RNase L pathways limit SINV replication in both human and bat cells. (A) DNA sequences encompassing the mutations from the *Mavs* gene of *bMAVS-*KO cells were amplified and sequenced and compared with the reference sequences of the gene. (B) WT and *bMAVS-*KO RoNi/7 cells were infected with SINV (MOI = 10 PFU/cell); at 12 h postinfection, cells were lysed; and RNA integrity was assessed on a Bioanalyzer. The positions of 18S and 28S rRNA are indicated. The data are from one representative of two independent experiments. (C) Cells were infected with SINV (MOI = 1 PFU/cell); at the indicated time points, the supernatants were harvested; and infectious viruses were titrated by plaque assay on Vero cells. The viral titer data are pooled from two independent experiments with three biological replicates in each experiment and expressed as means ± SDs (***, *P < *0.001; ns, not significant). (D) Cells were infected with SINV (MOI = 1 PFU/cell) or treated with 1,000 U of IFN-α; at 16 h after infection or treatment, cells were lysed; and proteins were analyzed by Western immunoblotting with rabbit polyclonal antibodies against bOAS3. The data in each panel are from one representative of two independent experiments. (E) WT and *bMAVS-*KO human A549 cells were infected with SINV (MOI = 1 PFU/cell); at 24 h postinfection, cells were lysed; and RNA integrity was assessed on a Bioanalyzer. The positions of 18S and 28S rRNA are indicated. The data in each panel are from one representative of two independent experiments. (F) Cells were infected with SINV (MOI = 1 PFU/cell); at the indicated time points, the supernatants were harvested; and infectious viruses were titrated by plaque assay on Vero cells. The viral titer data are pooled from two independent experiments with three biological replicates in each experiment and expressed as means ± SDs (***, *P < *0.001; ns, not significant).

To determine to what extent activation of RNase L is dependent on IFN signaling, we investigated the role of MAVS in OAS-RNase L activation. Thus, we used CRISPR/Cas9 editing to generate *bMAVS-*KO RoNi/7 cells. The KO was validated by sequencing (Fig. 8A) in the RoNi/7 *Mavs* gene. SINV infection induced rRNA degradation in *bMAVS-*KO as well as in control WT RoNi/7 cells (Fig. 8B), consistent with RNase L activation. While SINV replicated to more than 100-fold-higher titers in *bRNase L*-KO cells than in the parental WT cells (Fig. 6D and Fig. 8C), replication in *bMAVS-*KO cells did not show significant differences at 24 h postinfection and showed a slightly higher titer (3-fold) at 36 h postinfection (Fig. 8C). Similar data were obtained for activation of RNase L by rRNA degradation assay by Bioanalyzer (Fig. S4A) and for virus replication when a second *bMAVS-*KO clone was infected with SINV (Fig. S4B). While as expected KO of *MAVS* from RoNi/7 cells reduced bOAS2 and bOAS3 induction during SINV infection (Fig. 8D), there was no effect on the activation of RNase L. Similar results were observed when we used human A549 cells. During SINV infection, rRNA degradation was observed in *MAVS-*KO A549 cells (Fig. 8E). In addition, no significant titer differences were observed at 24 or 36 h postinfection in MAVS-KO A549 cells, while RNase L-KO cells showed 6-fold-higher titers than WT cells (Fig. 8F) at 24 h postinfection.

10.1128/mBio.02414-19.4 FIG S4 Comparison of RNase L activation levels of two MAVS-KO clones of RoNi/7 cells. (A) WT and two *bMAVS*-KO RoNi/7 clones were infected with SINV (MOI = 10 PFU/cell); at 12 hours postinfection, cells were lysed; and RNA integrity was assessed on a Bioanalyzer. The positions of 18S and 28S rRNA are indicated. The data are from one representative of two independent experiments. (D) WT and *bMAVS* RoNi/7 KO cells were infected with SINV (MOI = 1 PFU/cell); at the indicated time points, the supernatants were harvested; and infectious viruses were titrated by plaque assay on Vero cells. The data are pooled from two independent experiments with three biological replicates in each experiment and expressed as means ± SDs (ns, not significant). (The clones labeled “*#*1” are the ones used to generate the data in Fig. 8.) Download FIG S4, TIF file, 0.2 MB. Copyright © 2019 Li et al. 2019 Li et al. This content is distributed under the terms of the Creative Commons Attribution 4.0 International license.

We extended our findings to another virus, VACVΔE3L, a vaccinia virus mutant with a deletion of the gene encoding the E3L dsRNA binding protein; we previously showed that VACVΔE3L activates RNase L during infection of A549 cells (26). We infected WT and *bRNase L*-KO, *bOAS1*-KO, *bOAS2*-KO, *bOAS3*-KO, and *bMAVS-*KO cells with VACVΔE3L.

**FIG 9:**
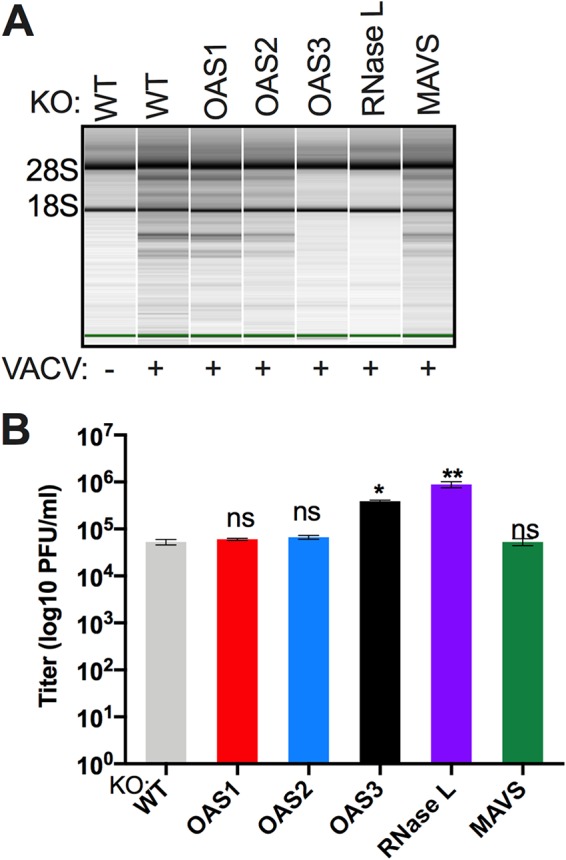
Activation of RNase L during vaccinia virus infection of RoNi/7 cells requires OAS3 expression and is independent of MAVS expression. (A) WT and KO RoNi/7 cells were infected with VACVΔE3L (MOI = 1 PFU/cell), and at 6 h postinfection cells were lysed and RNA integrity was assessed on a Bioanalyzer. The positions of 18S and 28S rRNA are indicated. The data are from one representative of two independent experiments. (B) Cells were infected in triplicate with VACVΔE3L (MOI = 1 PFU/cell); at 42 h postinfection, the cells were freeze-thawed three times; and infectious virus titers were determined by plaque assay on BHK-21 indicator cells. The viral titer data are from one of four independent experiments and expressed as means ± SDs (*, *P < *0.05; **, *P < *0.01; ns, not significant).

RNA was harvested from cells at 6 h postinfection for rRNA analysis. Degradation of rRNA, as assessed by Bioanalyzer, was observed in the infected WT, *bOAS1*-KO, *bOAS2*-KO, and *bMAVS-*KO cells but not in infected *bOAS3*-KO or *bRNase L*-KO cells (Fig. 9A). In addition, cells were lysed at 42 h postinfection for determination of viral replication by plaque assays. Consistent with the rRNA degradation data, viral titers were 17- and 7-fold higher in the *bRNase L-*KO or *bOAS3-*KO cells, respectively, than in the *bOAS1*, *bOAS2, bMAVS*, and WT cells (Fig. 9B). Thus, activation of RNase L during VACVΔE3L infection of bat RoNi/7 cells was dependent on RNase L and OAS3 but not OAS1, OAS2, or MAVS, similar to our findings with SINV.

## DISCUSSION

Since RNA viruses are often lethal in humans but relatively apathogenic in the natural bat host, it is important to understand host-virus interactions and how these may differ among hosts. We have focused on understanding expression and activation of the dsRNA-induced antiviral OAS-RNase L system, a pathway our group has investigated in detail in human and murine cells, but which had not been characterized in the bat. When we analyzed the annotated Egyptian Rousette genomic sequences in GenBank, we noted three genes, bOAS1, bOAS2, and bOAS3, homologous to human OAS genes, each with one or two ISREs in their promoter regions indicative of ISGs. We cloned and sequenced the open reading frames (ORFs) from the mRNAs encoding bOAS1, bOAS2, and bOAS3. Our findings are in contrast to the annotated Egyptian Rousette genomic sequences, which indicate two bOAS genes, *bOAS1*, which is predicted to encode bOAS1, a homologue of human OAS1, and a bOAS3-like gene which was predicted to encode a long OAS3-OAS2 fusion protein. Interestingly, OAS3-OAS2 fusion genes and proteins are predicted also by annotated GenBank sequences for two additional bat species, Eptesicus fuscus and Myotis brandtii, and three other nonbat species (donkey, ferret, and marmot). Using antiserum to detect OAS3 and OAS2 expression from bat cells, we did not detect a fusion protein for any of the bat species. It is likely that the OAS3-OAS2 fusion genes and resulting fusion protein predicted arise from annotation mistakes in GenBank.

In addition to the OAS genes, two OASL genes encoding OASL1 and OASL2 proteins were detected from genomic sequencing. Alignment of the bOAS and bOASL amino acid sequences with human and mouse protein sequences showed that the bat proteins are more similar to human homologues than to mouse proteins (Table 1 and Fig. 3). The crystal structure of human (31) and porcine (32) OAS1 has been well studied, and essential amino acids which correspond to dsRNA binding and 2-5A production have been identified. All three bOAS proteins contain highly conserved dsRNA binding amino acid residues as well as catalytic amino acids, suggesting that the bat OASs have the ability to recognize dsRNA and synthesize 2-5A. Bats encode bOASL1 and bOASL2, with homology to mouse and human OAS proteins. bOASL2 shares high sequence similarity with mouse OASL2 (see Fig. S2 in the supplemental material), suggesting that like the mouse protein, bOASL2 may have catalytic activity. In contrast, humans encode only one enzymatically inactive OASL protein. A recent study suggested that human OASL (enzymatically inactive) and mouse OASL2 (enzymatically active) suppress the function of cGAS-STING pathways (35, 36). This is surprising since hOASL is more similar to mouse OASL1 than to mouse OASL2 (Fig. S1). Nevertheless, it will be interesting to determine if bOASL1 or bOASL2 has a similar function. Xie et al. showed that a point mutation of STING from many bats species attenuates the function of STING (15). Thus, it is possible that bats may use two mechanisms to suppress the cGAS-STING pathway to make the host become more tolerant of viral infection.

Analysis of the promoter region of the bOAS genes revealed ISRE sequences in all the three genes, consistent with our qPCR data, showing that each bOAS mRNA is induced by IFN treatment and during viral infection (Fig. 1 and 2). Bat and human *Oas1* genes have one similar ISRE, and OAS1 mRNA expression is moderately inducible by IFN or virus infection (40- to 80-fold) in bats (Fig. 2), similar to our findings in human cells (26). Interestingly, while the *bOas2* gene has two adjacent ISREs and bOas2 mRNA expression is induced only 4- to 6-fold (Fig. 2), the human Oas2 has one ISRE and yet the expression of OAS2 mRNA is highly induced (10,000-fold) during infection of human cells (26). Similarly, while *bOAS3* has one ISRE, human *OAS3* has two ISREs, and OAS3 mRNA induction was about 9- to 15-fold in both bat and human cells with IFN. These findings suggest that the number of ISREs by itself does not have a major influence on the fold induction of OAS gene expression, with the caveat that extent of gene induction will vary with basal expression levels in different cell types. Interestingly, we detected basal OAS3 protein expression in RoNi/7 bat cells and basal OAS2 and OAS3 protein expression in Eptesicus fuscus skin fibroblasts. It is important to note that we cannot directly compare basal expression levels of OAS2 and OAS3 among cells from the various species of bats because the antisera were raised against RoNi/7 cell proteins and may preferentially react to the homologous proteins. Since we were unable to detect bOAS1 protein, it is possible, albeit unlikely, that OAS1 is not expressed in RoNi/7 cells. However, it is more likely that the OAS2 and OAS3 antibodies just do not cross-react with OAS1. This is not surprising as bOAS1 shares lower sequence homology (40%) with the catalytic domains of bOAS2 and bOAS3 than they do with each other. Importantly, further evidence that OAS1 protein is expressed is that the OAS1 gene is intact (Fig. 1), the mRNA is clearly expressed and induced by IFN or infection (Fig. 2) and contains an ORF encoding a protein very similar to human OAS1 with predicted active site intact (Fig. 3; Table 1), and OAS1-KO cells have increased SINV replication (Fig. 6).

In contrast to bat cells, we did not detect basal OAS protein expression in any human cells in our previous studies (Fig. 5A) (26, 37). While this may suggest that basal OAS could be important in enhancing bat resistance to viruses, it could also be due to the different cell types examined and differences in the sensitivity of detection of bat and human OAS proteins by Western blotting. There are clearly many more unanswered questions about the regulation of bOAS gene expression.

Infection of a series of RoNi/7 cells engineered using CRISPR/Cas9 to KO each *bOAS* gene and *bRNase L* showed that bOAS3 is essential for activation of RNase L during SINV or VACVΔE3L infection as assessed by rRNA degradation. (Please note that OAS2 is induced in *bOAS3-*KO cells as well as in WT cells.) These data are similar to our previous findings in several human cell lines, with diverse viruses. This was due to a higher affinity of OAS3 for long dsRNA compared to the shorter OAS1 and OAS2 proteins (21, 22), which is likely the case for the bat homologues as well. Also consistent with our previous findings in human cells, SINV replication is markedly increased (100- to 200-fold in the *bOAS3-*KO cells as well as *bRNase L*-KO cells compared to WT cells). Interestingly, in *bOAS1*-KO or *bOAS2*-KO cells SINV also replicates to a higher level than in WT cells, although not to the extent observed in *bOAS3-*KO or *bRNase L*-KO cells, and rRNA degradation was observed in cells lacking expression of either bOAS1 or bOAS2. These data suggest that bOAS1 and bOAS2 may exert some antiviral activity by activating RNase L in the absence of bOAS3. In human cells, we previously observed increased SINV replication in *OAS1-*KO cells at some time points. However, we observed rRNA degradation in *OAS1-*KO cells, as well as accumulation of 2-5A to the same extent as in WT human cells (26, 38), suggesting that this antiviral activity mediated by OAS1 in human cells may be at least in part independent of RNase L activation. Consistent with this observation, previous studies concluded that human OAS1 has an antiviral function which is RNase L independent (39). However, during infection of RoNi/7 cells with VACVΔE3L, as we previously found in human A549 cells, degradation of rRNA occurred to a similar extent in the absence of OAS1 and OAS2 as in WT cells and virus replicated to the same extent in *OAS1-*KO, *OAS2-*KO, and WT RoNi cells (Fig. 9), suggesting that only OAS3 played a role in antiviral activity. Further investigation needs to be carried out to understand the individual roles of OAS1 or OAS2 with different viruses and specifically whether there may be OAS-dependent antiviral activities independent of RNase L in bat or human cells.

OAS genes are ISGs, and as such, it has been generally thought that activation of RNase L is a downstream effect of IFN signaling. In this model, viral dsRNA would first need to be recognized by pattern recognition receptors (PRRs), such as RIG-I/MDA5, leading to IFN induction and subsequent upregulation of OASs, which would then be activated by viral dsRNA (40). However, we found previously that the OAS-RNase L pathway can be activated in the absence of murine coronavirus-induced IFN in mouse bone marrow-derived macrophages (41). Here, we show that activation of RNase L during infections with either SINV (Fig. 8) or VACVΔE3L (Fig. 9) is independent of MAVS expression in bat RoNi/7 cells, similar to findings for SINV in human A549 cells, indicating that basal OAS levels which are weakly induced in the absence of MAVS (Fig. 8D) are sufficient for activation of RNase L. We recently reported similar results during Zika virus infection of A549 cells (42). In addition, MAVS plays little if any role in limitation of SINV replication in bat or human cells (Fig. 8B and E) or of VACVΔE3L in bat cells (Fig. 9B). This is in contrast to RNase L, which, as discussed above, dramatically limits viral replication (Fig. 6, 8, and 9) (26). These results imply that the OAS-RNase L pathway plays a significant role in limiting viral infection before IFN induction or in the absence of IFN by a virus that antagonizes IFN induction or signaling. Indeed, a recent study showed robust RNase L activation as early as 2 h after pIC transfection in human A549 cells (43). Thus, whereas IFN induction of OASs may enhance the activation of the pathway, it is not always required. These studies suggest that OASs act in parallel to other PRRs, including RIG-I-like receptors, and the OAS-RNase L system is a separate pathway rather than a secondary pathway of IFN induction/signaling. Importantly, OAS-RNase L is a potent antiviral pathway, activated by infection in bat RoNi/7 cells.

## MATERIALS AND METHODS

### Cells and viruses.

Egyptian Rousette (Rousettus aegyptiacus) kidney-derived RoNi/7 cells (44–46) were obtained from Marcel Müller (Charité-Universitätsmedizin, Berlin, Germany), and Myotis lucifugus skin fibroblasts (47), Myotis lucifugus embryonic fibroblasts, *Myotis velifer* embryonic fibroblasts, and Eptesicus fuscus skin fibroblasts (47) were obtained from Cedric Feschotte (Cornell University). The cells were cultured in Dulbecco’s modified Eagle’s medium (DMEM; Gibco catalog no. 11995) supplemented with nonessential amino acids, 10% fetal bovine serum (FBS), 100 U/ml of penicillin, and 100 mg/ml streptomycin. African green monkey kidney Vero cells (ATCC CCL81) were cultured in DMEM (Gibco 11965), supplemented with 10% FBS, 10 mM HEPES, 1 mM sodium pyruvate, 100 U/ml of penicillin, 100 mg/ml streptomycin, and 50 μg/ml gentamicin. Baby hamster kidney (BHK-21) cells were cultured in DMEM supplemented with 10% FBS, 5% tryptose phosphate broth (Sigma-Aldrich), 100 U/ml penicillin, and 100 mg/ml streptomycin. Human HEK 293T cells were cultured in DMEM supplemented with 10% FBS and 1 mM sodium pyruvate. Human A549 cells were cultured in RPMI 1640 medium (Gibco) supplemented with 10% fetal bovine serum (FBS), 100 U/ml of penicillin, and 100 mg/ml streptomycin. Human HEK 293T and A549 cells have been authenticated by the ATCC. The RoNi/7 cells were authenticated by cloning and sequencing of OAS and RNase L mRNAs, which matched the sequences derived from the Egyptian Rousette (*Rousettus aegyptiacus*) bat genes deposited in GenBank.

RNase L or MAVS-KO A549 cells were generated by CRISPR/Cas9 technology and were described in previous studies (26, 38). Sindbis virus Girdwood G100 (SINV), expressing mCherry, was obtained from Mark Heise (University of North Carolina, Chapel Hill) and was prepared in BHK cells as previously described (48). Sendai virus (SeV) strain Cantell (49) was obtained from Carolina B. Lopez (University of Pennsylvania). Mutant vaccinia virus (VACVΔE3L) was obtained from Bertram Jacobs (Arizona State University, Tempe, AZ) and was grown as described previously (50).

### Cloning and sequencing of bat OAS cDNAs.

bOAS1, bOAS2, and bOAS3 cDNAs were cloned from RoNi/7 cells. Briefly, the cells were treated with 1,000 U of IFN-α overnight, and total cellular RNA was extracted using an RNeasy Plus Mini kit (Qiagen). cDNA was synthesized by reverse transcription using SuperScript III (Invitrogen) and mRNA-specific reverse primers. bOAS1-rev and bOAS2-rev (Table 1) primers were used for reverse transcription to synthesize bOAS1 and bOAS2 cDNA, respectively. Three cDNA fragments were cloned to assemble the full length of the bOAS3 open reading frame. bOAS3-F1-rev, bOAS3-F2-rev, and bOAS3-F3-rev (Table 1) were used for reverse transcription reactions to synthesize three cDNA fragments (bOAS3-F1, bOAS3-F2, and bOAS3-F3, respectively). The DNA fragments of bOAS1, bOAS2, bOAS3-F2, and bOAS3-F3 were amplified by using AccuPrime DNA polymerase (Invitrogen) with the forward primers and reverse primers (see Table S1 in the supplemental material), and were cloned into pCR2.1 TOPO vectors (Invitrogen). The DNA fragment of bOAS3-F1 was amplified by using Q5 DNA polymerase (NEB) and was cloned into pCR II Blunt-TOPO vectors (Invitrogen). The cloned DNAs were sequenced and analyzed. The OAS1 sequence matched the predicted mRNA sequence in GenBank (ID 107513273).

10.1128/mBio.02414-19.5 TABLE S1 Primers for cloning and sequencing of bOAS1, bOAS2, and bOAS3 cDNAs. Download Table S1, DOCX file, 0.02 MB. Copyright © 2019 Li et al. 2019 Li et al. This content is distributed under the terms of the Creative Commons Attribution 4.0 International license.

### Construction of plasmids and pseudolentivirus.

The oligonucleotide sequences to be used for generation of small guide RNAs (sgRNAs) for KOs of *bOas1*, *bOas2*, *bOas3*, *bRNase L*, and *bMavs* genes are shown in Table S2. A pair of forward and reverse oligonucleotides for generation of each sgRNA (synthesized by IDT) were annealed by published methods (51) and were inserted into pLenti-CRISPR (Addgene) between BsmBI restriction sites. The resulting plasmids are named pLenti-sgbO1 (targeting the *bOas1* gene), pLenti-sgbO2 (targeting the *bOas2* gene), pLenti-sgbO3 (targeting the *bOas3* gene), pLenti-sgbRL (targeting the *bRNase L* gene), and pLenti-sgbMA (targeting the *bMavs* gene).

10.1128/mBio.02414-19.6 TABLE S2 Construction of the plasmids for knockout of bat *Oas1*, *Oas2*, *Oas3*, *RNase L*, and *Mavs* genes by CRISPR/Cas9 technology. Download Table S2, DOCX file, 0.02 MB. Copyright © 2019 Li et al. 2019 Li et al. This content is distributed under the terms of the Creative Commons Attribution 4.0 International license.

For packaging of pseudolentiviruses, 1 × 10^6^ HEK 293T cells were plated in one well of a 6-well plate, and the next day were transfected with 5 μg pLenti-CRISPR (with sgRNA), 3.5 μg psPAX2, and 1.25 μg of pCMV-VSV-G (obtained from Paul Bates, University of Pennsylvania) using Lipofectamine 2000 (Invitrogen) (24 μl in 250 μl of DMEM). The supernatants were harvested at 24 and 48 h posttransfection and stored at −80°C, and the 48-h supernatants were used for further KO experiments.

### Construction of *bOas1*, *bOas2*, *bOas3*, *bRNase L*, and *bMavs* gene knockout RoNi/7 cells.

For construction of RoNi/7 KO cells using Lenti-CRISPR, 2 × 10^5^ cells were plated into one well of a 24-well plate and were transduced with 250 μl of pseudolentiviruses. Forty-eight hours postransduction, cells were cultured in medium containing 0.5 μg/ml of puromycin for 3 days. The resistant cells were further cultured for 1 week in medium without puromycin before being cloned by limited dilution. Briefly, cells were diluted to 10 cells/ml and 200 μl of cells was added to one well of 96-well plates. Single cells were selected for further amplification and genotyping.

Genomic DNA was extracted using a DNeasy Blood and Tissue kit (Qiagen). DNA fragments covering the region targeted by sgRNA were amplified by PCR using gene-specific primers (Table S3) mixed with GoTaq master mix (Promega), and the PCR products were sequenced. Cells with frameshift mutations (deletion or insertion) in targeted genes were selected for further experiments.

10.1128/mBio.02414-19.7 TABLE S3 Primers for genotyping of *Oas1*, *RNase L*, and *Mavs* knockout cells. Download Table S3, DOCX file, 0.02 MB. Copyright © 2019 Li et al. 2019 Li et al. This content is distributed under the terms of the Creative Commons Attribution 4.0 International license.

### Generation of 3×FLAG-hOAS3 knock-in cells.

bOAS3-KO cells were electroporated (BTX) with plasmid pCMV10-3XFLAG-hOAS3 (26) and plated without antibiotics. At 48 h postelectroporation, cells were selected with 250 μg/ml of G418 (Gibco) for 3 weeks and then cloned by limiting dilution. Clones were screened by Western immunoblotting using anti-FLAG M2 antibodies. Cell clones expressing 3×FLAG-hOAS3 were maintained in medium containing 125 μg/ml of G418.

### Antibody.

Rabbit anti-bOAS2 and -bOAS3 antibodies were generated by immunizing rabbits with purified MBP-bOAS2-D2 and MBP-bOAS3-F2/3. Briefly, bOAS2 domain 2 (catalytic domain) was amplified by PCR using primers bOAS2-D2-for (ACGTCGACTACCCCCGGGCATCTTCTGGATAAATTC) and bOAS2-D2-rev (GCTCTAGATCACTCGAGGAGCCCCCAACTTCTGAAC), and bOAS3 fragment F2-F3 was amplified by PCR using primers bOAS3-F2/3-for (ACGTCGACTGATCTGTCTCAGATCCCCGCCAATGAG) and bOAS3-F2/3-rev (GCCAAGCTTTCAGACGTGTCCAAGGGGAGGGACCACATG).

The fragments were cloned into pMal (NEB) vectors between SalI and XbaI restriction sites. pMal-bOAS2-D2 and pMal-bOAS3-F2/3 were transformed into NEB-Express competent Escherichia coli, and IPTG was added to the bacterial cultures to induce expression of the proteins. The proteins were purified by elution from the protein bands cut from polyacrylamide gels with the Pierce zinc reversible stain kit (Thermo Fisher). The eluted proteins were concentrated and showed a single band on a polyacrylamide gel by staining. Rabbits were injected with 250 μg protein each 4 times over about 2 months before bleeding. Mouse anti-GAPDH GA1R (1:1,000; Thermo Fisher) was used to detect bat GAPDH. Mouse anti-FLAG M2 antibodies (1:1,000; Sigma-Aldrich) were used to detect 3×FLAG-hOAS3. Secondary antibodies goat anti-mouse antibody (1:5,000; Santa Cruz) and goat anti-rabbit antibody (1:3,000; Cell Signaling), conjugated to horseradish peroxidase (HRP), were used to detect mouse- or rabbit-derived primary antibodies.

### Western blotting.

Confluent cells in 12-well plates were treated or mock treated with 1,000 U of IFN-α overnight or infected with SINV at an MOI of 1 at 16 h postinfection. Cells were harvested, washed in PBS, and lysed with NP-40 buffer with protease inhibitor cocktail (Roche). Cell lysates were mixed with 4× Laemmli buffer, boiled at 95°C for 5 min, and analyzed by electrophoresis on 4 to 12% or 4 to 15% gradient SDS gels. Proteins were transferred to polyvinylidene difluoride membranes, which were treated with 5% nonfat milk in TBST (Tris-HCl-buffered saline with 0.5% Tween 20) blocking buffer for 1 h, followed by incubation overnight at 4°C with antibodies diluted into TBST. Membranes were then washed three times with TBST, incubated with secondary antibodies for 1 h at room temperature, again washed three times with TBST, and then incubated with SuperSignal West Dura extended-duration substrate (Thermo Fisher), and the signal was detected using an Amersham Imager 600 (GE).

### Virus growth kinetics.

Cells were plated in 12-well (RoNi/7) or 6-well (A549) plates, 1 × 10^6^ cells per well. The next day, cells were infected with the indicated viruses at an MOI of 1, three parallel wells per virus. At 6, 24, and 36 h postinfection, 200 μl of supernatant was harvested and stored at −80°C for titration.

### Plaque assays.

SINV was diluted in DMEM, and 200 μl was added to confluent Vero monolayers in 6-well plates. The plates were incubated for 1 h at 37°C and were rocked at 15-min intervals. Cells were then overlaid with 3 ml warm DMEM containing 1% FBS and 0.7% agar. Vaccinia virus (VACVΔE3L) titers were determined in BHK-21 cells as described previously (50).

### rRNA cleavage assay.

Cells were infected with SINV at an MOI of 10 (bat cells) or an MOI of 1 (A549 cells). At 12 (bat cells) or 24 (A549 cells) h postinfection, cells were harvested in RLT buffer (RNeasy minikit; Qiagen). Bat cells were infected with VACVΔE3L at an MOI of 1 and at 6 h postinfection were harvested in RLT buffer (Bio Basic, Inc.). Total RNA was extracted and was resolved on RNA chips using an Agilent 2100 Bioanalyzer (52).

### mRNA quantification by quantitative reverse transcriptase PCR (qRT-PCR).

Cells were infected with SINV (MOI = 10) or SeV (MOI = 10) or treated with 1,000 U of IFN-α in triplicate in 12-well plates. Cells were lysed at 12 (SINV) or 24 (SeV) h postinfection (hpi) or 24 h after IFN treatment in RLT Plus RNA lysis buffer (Qiagen), and RNA was isolated using the RNeasy Plus Mini kit (Bio Basic, Inc.) as previously described (52). Cycle threshold (*C_T_*) values were normalized to β-actin. OAS mRNA levels were calculated relative to β-actin mRNA and expressed as fold over mock-treated or mock-infected with the formula 2^−Δ(Δ^*^CT^*^)^ (Δ*C_T_* = *C_T_*_gene of interest_ − *C_T_*_actin_). Primer sequences are listed in Table S4 in the supplemental material.

10.1128/mBio.02414-19.8 TABLE S4 qRT-PCR primers for bat *actin*, *Oas1*, *Oas2*, *Oas3*, *RNase L*, and *Ifit1* genes. Download Table S4, DOCX file, 0.02 MB. Copyright © 2019 Li et al. 2019 Li et al. This content is distributed under the terms of the Creative Commons Attribution 4.0 International license.

### Software and statistical analysis.

Sequences were analyzed by Vector NTI. Sequence alignment figures were created by Vector NTI. All other figures were created by GraphPad. All analyses were performed in GraphPad Prism version 7.0a. Plaque assay data were analyzed by two-way ANOVA. Significance is shown as follows: NS, not significant; *, *P* < 0.05; **, *P* < 0.01; ***, *P* < 0.001; ****, *P* < 0.0001.

### Data availability.

The nucleotide sequences of bOAS2 and OAS3 ORFs were deposited in GenBank with accession numbers MK392547 and MK392548, respectively.
