# Supplementary figures and images for: Activation of RNase L in Egyptian Rousette Bat-Derived RoNi/7 Cells Is Dependent Primarily on OAS3 and Independent of MAVS Signaling
Source: mBio. 2019 Nov 12;10(6):e02414-19. doi: 10.1128/mBio.02414-19 (PMC6851283; doi:10.1128/mBio.02414-19)

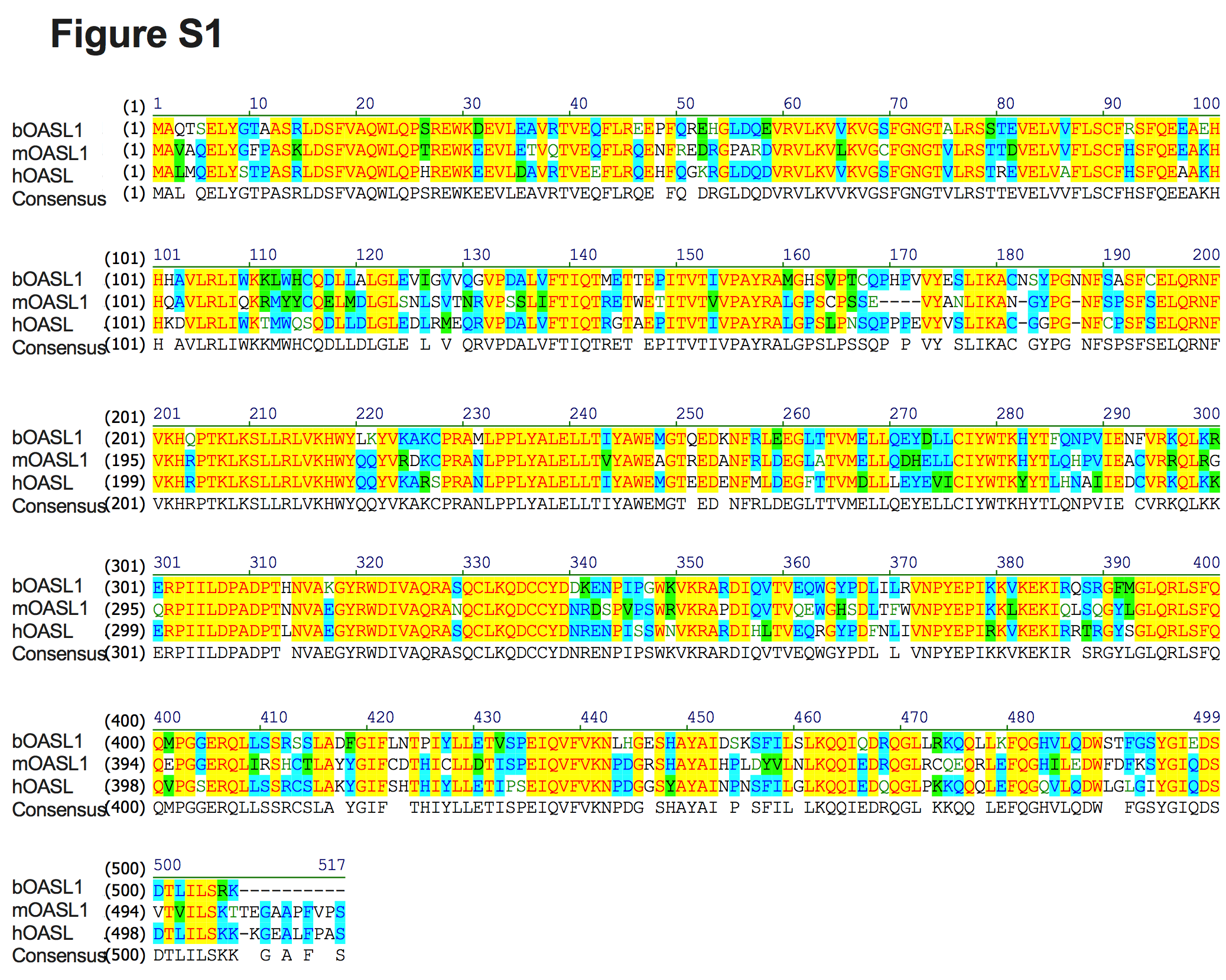

Supplement: FIG S1 [file mBio.02414-19-sf001.tif]

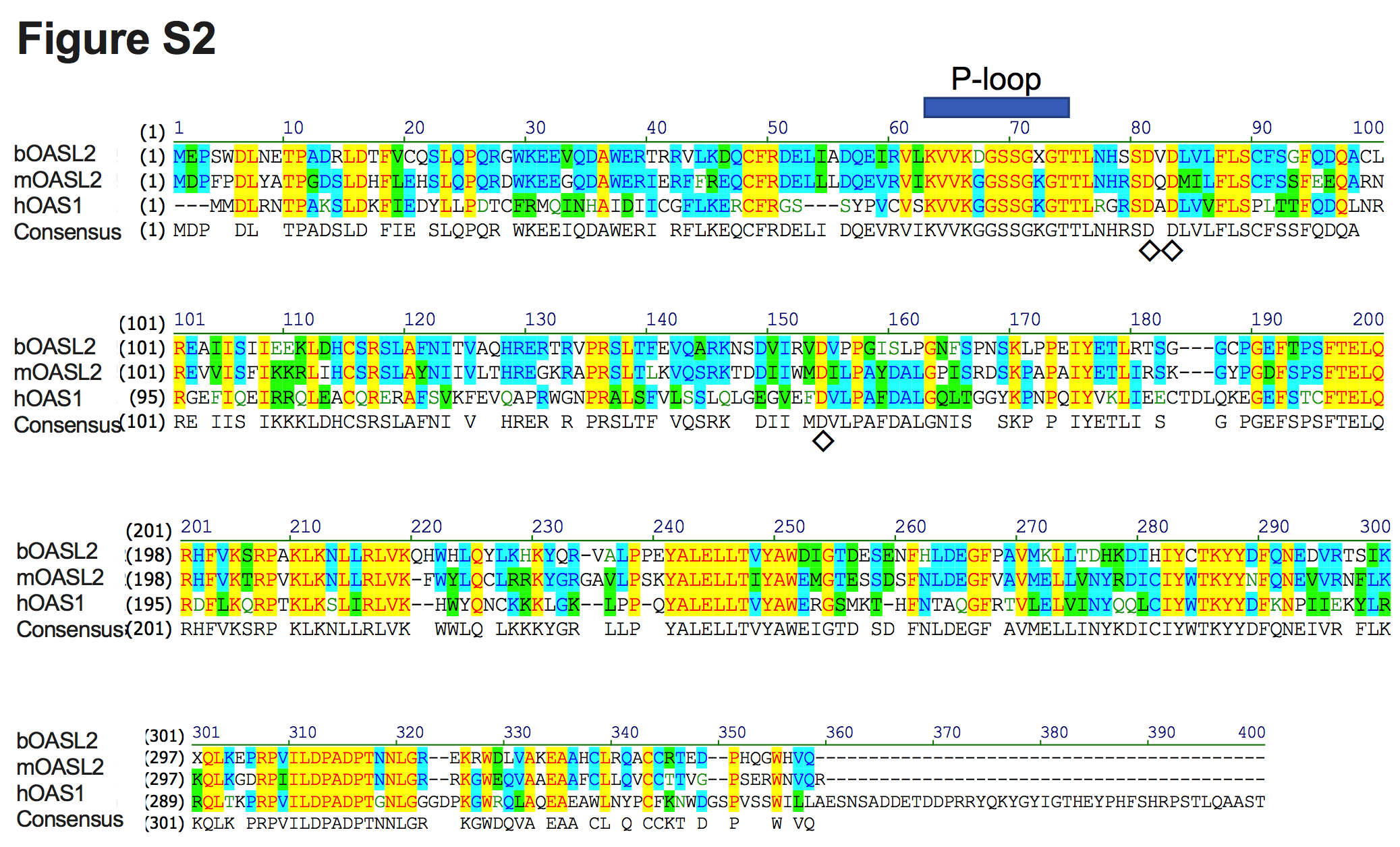

Supplement: FIG S2 [file mBio.02414-19-sf002.tif]

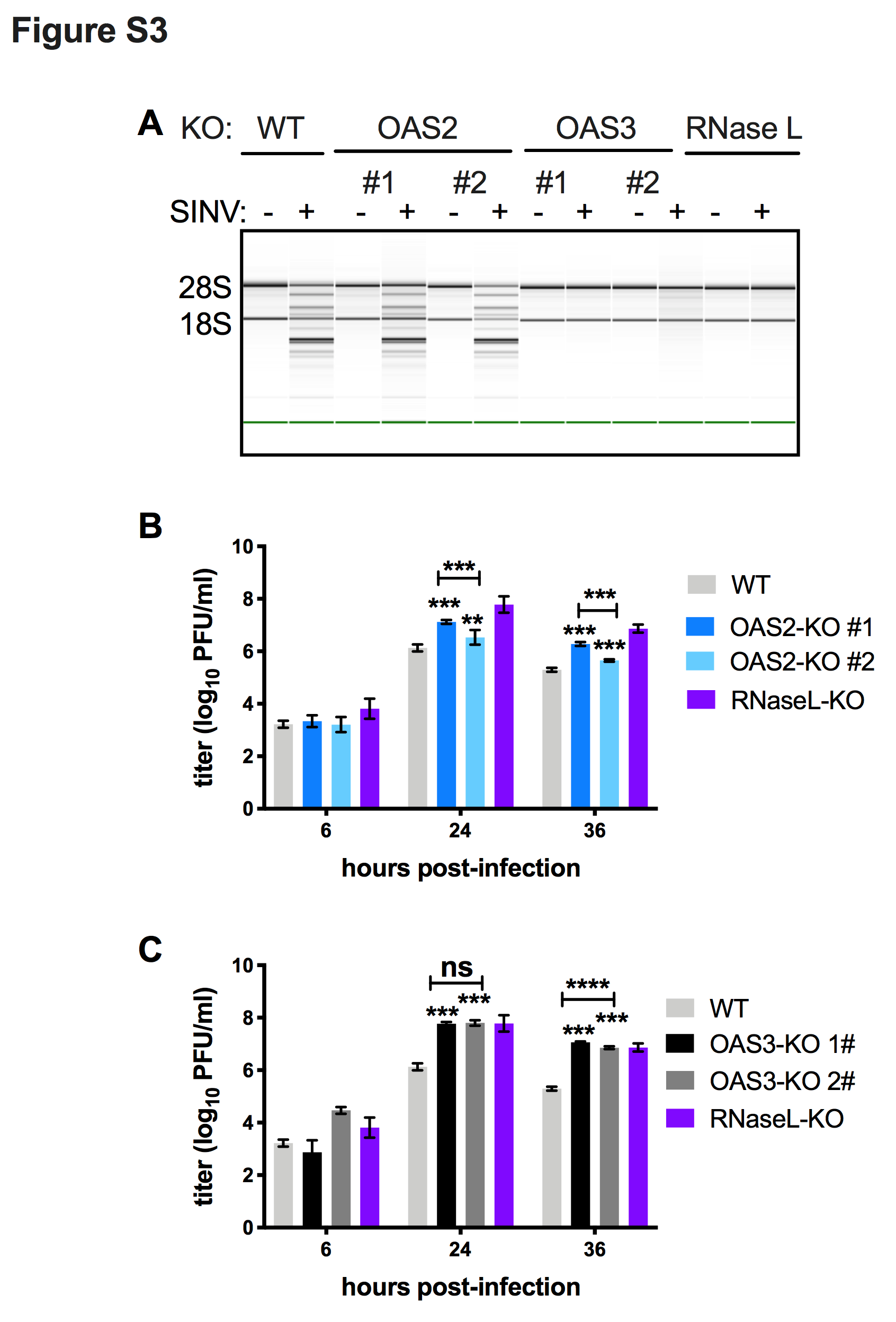

Supplement: FIG S3 [file mBio.02414-19-sf003.tif]

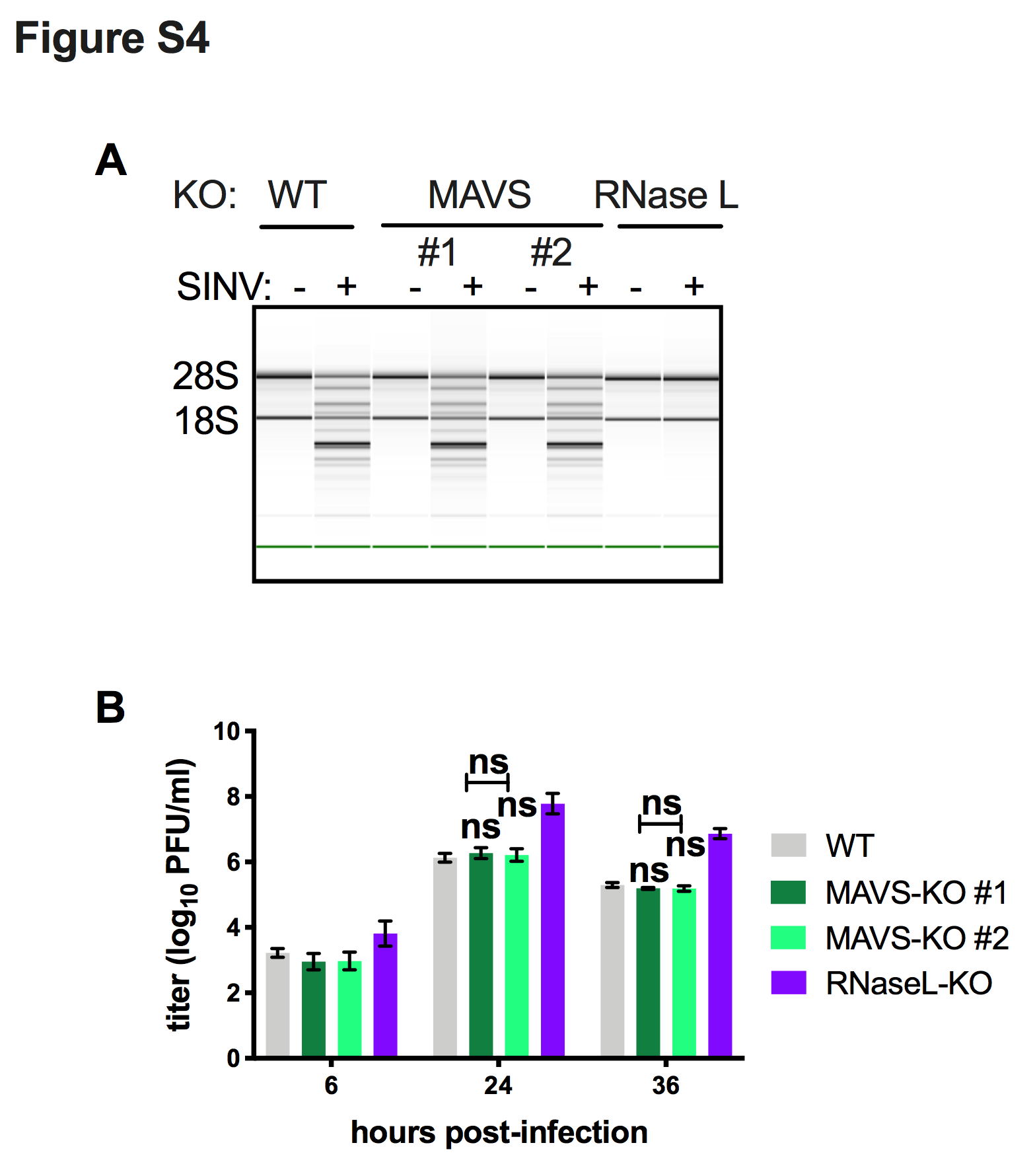

Supplement: FIG S4 [file mBio.02414-19-sf004.tif]
