# Supplementary material for: Activation of RNase L in Egyptian Rousette Bat-Derived RoNi/7 Cells Is Dependent Primarily on OAS3 and Independent of MAVS Signaling
Source: mBio. 2019 Nov 12;10(6):e02414-19. doi: 10.1128/mBio.02414-19 (PMC6851283; doi:10.1128/mBio.02414-19)
Supplement: TABLE S1 [file mBio.02414-19-st001.docx]

Table S1. Primers for cloning and sequencing of of bOAS1, bOAS2, bOAS3 cDNAs.

| cDNA | Primers | Nucleotides Sequences (5’-3’) |
| --- | --- | --- |
| bOAS1 | bOAS1-for | ATGGATCTCTCCAAAATCCCAGCTAG |
|  | bOAS1-rev | GAGGATGACACACCACTGGTTCTC |
| bOAS2 | bOAS2-for | ATGGGAAACTACCTGCTGTACCGGAAG |
|  | bOAS2-rev | CTCGAGGAGCCCCCAACTTCTGAACG |
| bOAS3 | bOAS3-F1-for | ATCGATATGGATGTGTACCGCACCCCCGCG |
|  | bOAS3-F1-rev | GATCTGAGACAGATCTGGCGTAGAAG |
|  | bOAS3-F2-for | CTTCTACGCCAGATCTGTCTCAGATC |
|  | bOAS3-F2-rev | ACTTTGATGGGGGAATTCCGGAAGCAG |
|  | bOAS3-F3-for | CTGCTTCCGGAATTCCCCCATCAAAGT |
|  | bOAS3-F3-rev | GGTGTCCGGTGACGTGTCCAAGGGGAGGG |
|  |  |  |
