# Supplementary material for: Activation of RNase L in Egyptian Rousette Bat-Derived RoNi/7 Cells Is Dependent Primarily on OAS3 and Independent of MAVS Signaling
Source: mBio. 2019 Nov 12;10(6):e02414-19. doi: 10.1128/mBio.02414-19 (PMC6851283; doi:10.1128/mBio.02414-19)
Supplement: TABLE S2 [file mBio.02414-19-st002.docx]

Table S2. Construction of the plasmids for knockout of bat *Oas1,* *Oas2*, *Oas3,* *Rnasel* and *Mavs* genes by CRISPR/Cas9 technology

| Genes | Primers | Nucleotides Sequences (5’-3’)* |  |
| --- | --- | --- | --- |
| *bOas1* | sgbO1 Forward | CACCG*CAGATCTCTAGCTGGGATTT* |  |
|  | Reverse | AAAC*AAATCCCAGCTAGAGATCTG*C |  |
| *bOas2* | sgbO2 Forward | CACCG*GGAGTTGGATACCTTTATCC* |  |
|  | Reverse | AAAC*GGATAAAGGTATCCAACTCC*C |  |
| *bOas3* | sgbO3 Forward | CACCG*GATCAGGTTCTTCAGCTTGG* |  |
|  | Reverse | AAAC*CCAAGCTGAAGAACCTGATC*C |  |
| *bRnase L* | sgbRL Forward | CACCG*CAGCCGTAAATATAGATAAG* |  |
|  | Reverse | AAAC*CTTATCTATATTTACGGCTG*C |  |
| *bMavs* | sgbMA Forward | CACCG*GAATCTCCAGAACATGAATA* |  |
|  | Reverse | AAAC*TATTCATGTTCTGGAGATTC*C |  |
|  |  |  |  |
|  |  |  |  |

* Nucleotides sequences target the genes were indicated as italic letters.
