# Supplementary material for: Activation of RNase L in Egyptian Rousette Bat-Derived RoNi/7 Cells Is Dependent Primarily on OAS3 and Independent of MAVS Signaling
Source: mBio. 2019 Nov 12;10(6):e02414-19. doi: 10.1128/mBio.02414-19 (PMC6851283; doi:10.1128/mBio.02414-19)
Supplement: TABLE S3 [file mBio.02414-19-st003.docx]

Table S3. Primers for genotyping of *Oas1*, *Rnasel* and *Mavs* knockout cells.

| Genes | Primers | Nucleotides Sequences (5’-3’) |
| --- | --- | --- |
| *bOas1* | *bOas1* Forward | TTCTGGCAGCAGAAGAGGAAG |
|  | Reverse | ATGGCCTAGGGCGGTGGTTC |
| *bRnasel* | bRL Forward | TCACGTGGACAGCAGGAAGT |
|  | Reverse | CGCATTAATCATCAAACTGA |
| *bMavs* | bMA Forward | GTGTTTTGTCCAACCCCACGTCC |
|  | Reverse | GGACGTGGGGTTGGACAAAACAC |
|  |  |  |
