# Supplementary material for: Activation of RNase L in Egyptian Rousette Bat-Derived RoNi/7 Cells Is Dependent Primarily on OAS3 and Independent of MAVS Signaling
Source: mBio. 2019 Nov 12;10(6):e02414-19. doi: 10.1128/mBio.02414-19 (PMC6851283; doi:10.1128/mBio.02414-19)
Supplement: TABLE S4 [file mBio.02414-19-st004.docx]

Table S4. qRT-PCR primers for bat *actin, Oas1, Oas2, Oas3, Rnasel L and Ifit1* genes.

| Genes | Forward (5’-3’) | Reverse (5’-3’) |
| --- | --- | --- |
| *bOas1* | GGAGGGCGAGTTCTC | CCCAAGCATAGACCGTCAGG |
| *bOas2* | TCCGAAGACCTCAACGAAAG | CGGGAGAGGACGAAGT |
| *bOas3* | CGCCCTCAGGTCTACGTG | TCCCCTTGGGCATCTTGTTG |
| *bRNasel* | TCATCATCGCTGGGATCGT | CTCCGCTCCATTCTCATACA |
| *bIfit1* | GTCATCACCATTGGCAACGA | CGTGTTGGCGTAGAGGTC |
| *bactin* | CCTGAAGCTTCAGGATG | CCCTATCTGGTGATGCAG |
